# Supplementary material for: Who enjoys solitude? autonomous functioning (but not introversion) predicts self-determined motivation (but not preference) for solitude
Source: PLoS One. 2022 May 25;17(5):e0267185. doi: 10.1371/journal.pone.0267185 (PMC9132342; doi:10.1371/journal.pone.0267185)
Supplement: Attachment — Submitted filename: Review for PONE.docx [file pone.0267185.s001.docx]

Review for PONE-D-21-07216 Who Enjoys Solitude? Autonomous Functioning (But Not Introversion) Predicts Self- Determined Motivation (But Not Preference) for Solitude

May 18, 2021

The authors have contributed a valuable addition to the empirical literature on solitude, which is sparse. In particular, they tackle the issue of differentiating the construct of solitude from the individual difference of introversion, which as they note has often been conflated, with mixed results from previous studies. In addition, their study asserts the importance of dispositional autonomy as a factor in solitude enjoyment, a factor that has largely been overlooked in the solitude literature. I have organized my comments according to the sections of the manuscript.

Introduction:

1. A correction on Thomas & Azmitia’s (2019) findings on p. 5. The authors state that Burger’s Preference for Solitude (PSS) measure showed negative correlation with self-determined motivation for solitude, but in actuality their results showed a significant positive correlation for both emerging adults and adolescents. The PSS also correlated positively with not-self-determined motivation, indicating that the PSS does not differentiate between intrinsic/autonomous/self-determined vs. extrinsic/non-autonomous/not-self-determined motivations.

2. Please cite the study in this sentence (p. 5): *“In contrast, in another study that examined preference for solitude by Burger’s operationalization and self-determined motivation for solitude from SDT perspective in older adults showed positive correlations between these two concepts.”*

3. It should be noted that Burger also found a significant positive correlation between introversion and preference for solitude in his original study (1995).

4. Can the authors state their hypothesis more clearly in the following sentence (p. 7): *“We predicted that extraversion-introversion dimension of big-five personality would positively relate to preference for solitude but yield non-significant association with self-determined motivation for solitude.”*

The phrase “extraversion-introversion dimension” renders the hypothesis unclear. Based on their introduction, I understand them to mean that introversion will positively correlate with preference for solitude; however, the Big 5 measures extraversion (high or low), thus a positive correlation would indicate that extraverts prefer solitude. It could be restated that extraversion scores will negatively correlate with preference for solitude (and motivation for solitude). Or, clearly state that you are reverse scoring the extraversion measure, so that all extravert items are reverse coded to yield an introvert score. You mention this briefly in the Methods section, but it is so non-traditional to frame this factor as Introversion that I think it would be worth explaining more clearly to the reader. On a related note, it is troubling that introversion on the Big 5 is so negatively valenced (defined with the items of shy, reserved, inhibited, as well as low scores on all of the “positive” items). I don’t know if this affects the authors’ interpretation of their findings, but it could be worth noting (there is a long tradition of such critiques of the Big 5 for this, and other reasons, e.g. Block 1995; 2010). This is not the authors’ problem, but it could be worth mentioning as a possible explanation for why laypeople and researchers alike regularly “intuit” that introversion must be related to solitude. Perhaps it is because the Big 5 is not accurately measuring introversion – only the negative side of low extraversion.

5. To support the authors’ framing of mixed results when it comes to introversion and solitude, the authors may want to include a more recent study by Thomas and colleagues (2020), in which cluster analysis differentiated between two clusters of introverts (high-functioning and low-functioning re: measures of adjustment), and these clusters did show different associations to preference for solitude, loneliness, and motivation for solitude.

6. The rationale for their second hypothesis (dispositional autonomy) is clear and well-reasoned.

Study 1

7. I appreciate that the authors utilized a daily diary methodology for this study, as I agree these types of methods improve ecological validity in general, and in particular for assessing how people experience their time alone.

8. A minor clarification - In Weinstein et al’s original study, the measure is called the Index of Autonomous Functioning (IAF) whereas the authors of this manuscript use the term Autonomous Functioning Index (AFI). I am not sure if the name of the scale has been altered since its original publication, or whether this is an error. Please revert to IAF if necessary.

9. A couple of comments on their preference for solitude measure. I found it odd that the authors did not use Burger’s Preference for Solitude Scale in their study after discussing it in their introduction. It can be difficult to compare results between this study and prior studies if the measures are not the same. It would be helpful if the authors could include an explanation for why his original 12-item scale was not used in their initial survey, along with the other personality measures. Second, it would be helpful if the authors could briefly explain how they selected the three questions they did use to assess preference for solitude during the daily diary study. For example, were their three questions inspired by, or derived from, Burger’s measure in any way? Were they theoretically generated based on other sources?

10. Please include the rating scale used for preference for solitude. Was it 1-5? This would help readers interpret the descriptive statistics on p. 13.

11. As with point 9 above, it would be helpful to briefly explain how they selected the four questions used to assess self-determined motivation for solitude during the daily diary study.

12. In the daily diary prompts, were the self-determined motivation questions only presented to participants if they had spent time alone that day? The way these questions are worded, it sounds as if they are referencing actual time the participant had spent alone.

13. Did the study collect data on the frequency or duration of time spent alone each day? If so, that would be valuable to add into the analysis.

Study 2

14. The inclusion of all 5 factors of the Big 5 Inventory is presented, convincingly, as a worthwhile addition in order to evaluate how influential dispositional autonomy is for predicting solitude behaviors.

15. It could be worth noting that agreeableness and conscientiousness were both marginally significant (<.10). They are very close to the .05 cutoff, and very close to the introversion results. In particular the results show a trend with low agreeableness being associated with preference and motivation for solitude. Theoretically this might make sense, given that highly agreeable people may be less able to resist social pressure (one of the aspects of the IAF), so people lower in agreeableness could potentially find it easier to withdraw from the social scene (potentially disrupting social norms or social harmony) and take their alone time.

Discussion

16. Change personality *types* to *traits.* (p. 20).

17. The stronger case for self-determined motivation for solitude and positive development was found with emerging adults rather than adolescents in the Thomas & Azmitia study they reference (p. 20).

18. I found the section (p. 21-22) following this sentence particularly strong and theoretically sound: *In sum, the associations between dispositional autonomy and seeing time alone as enjoyable and valuable demonstrated the first evidence to show that solitary enjoyment related to the ability to regulate oneself in positive and self-congruent way.*

19. Just a side note, re: a statement on p, 23 “…*it is possible that the same personality characteristics that lead people to enjoy their time with others also predict their affinity for solitude.”* Maslow made the essentially same point in his studies of self-actualization, when he noted that self-actualized people have both a love of privacy and a love of other people.

20. On p. 23, *“To date, we are aware of no empirical data that has directly assessed the link between any personality characteristics…”* but didn’t both Burger (1995) and Thomas and colleagues (2019; 2020) include introversion-extroversion data in relation to preference for solitude and motivation for solitude? See points 3 and 5, above. But, to the authors’ point, this study does appear to be the first that shows the links between the personality characteristic of autonomous functioning and solitude.

Proofreading:

The authors will want to proofread for typos, as there were many in the manuscript. A few examples, although I didn’t document them all: “I do *thinks* in order to avoid feeling ashamed” (p. 11); “Sixteen participants who filled out the initial survey *did not the* diary portion of the study…” (p. 16); “ First, *all two* studies relied on” (p. 23); etc.

Figures 1 and 2 have the same title. Can the authors add “in Study 1” to Figure 1 and “in Study 2” in Figure 2 (or something to that effect) so that the figures are differentiated?
