# Supplementary material for: Who enjoys solitude? autonomous functioning (but not introversion) predicts self-determined motivation (but not preference) for solitude
Source: PLoS One. 2022 May 25;17(5):e0267185. doi: 10.1371/journal.pone.0267185 (PMC9132342; doi:10.1371/journal.pone.0267185)
Supplement: Attachment — Submitted filename: Responses to editor and reviewers comments.docx [file pone.0267185.s002.docx]

**Sample size justification**

**The Editor commented:**

“We believe it is justified to determine sample sizes based on realistic measures: expectations of how many participants we can recruit from the pool and lack of external funding to recruit outside of this pool.” This explanation is inadequate. Consider a study that drew from a very small pool of participants and had no funding support, permitting a recruitment of 10 people. Would this be sufficient justification for that sample size? I think not. If your studies are not appropriately powered the reader doesn't know how reliable the findings are. This casts your study findings into considerable doubt. The lack of power analyses for both studies, and the consequences thereof, should be clearly acknowledged in your General Discussion.

**Response:** Since I did not run power analysis to determine our sample sizes for Study 1 and Study 2, I conducted power simulations to examine what is the smallest detectable effect size, given multiple possible sample sizes. I have now added this in the Discussion on page 24: “Since we did not conduct power analyses to determine our sample sizes for both studies, we conducted power calculation on simulated data. This is more appropriate than performing post-hoc power analyses, which have been criticized for not providing true observed power when being conducted on data that has been collected and analysed (47). In a series of power simulations (n = 100 simulations) looking at achieved power across sample sizes between 140 and 270 and assuming 5 to 7 data points per participant, a sample of 270 (Study 2’s sample) should allow 80% power to detect an effect size as small as .08.”

Reference: Zhang Y, Hedo R, Rivera A, Rull R, Richardson S, Tu XM. Post hoc power analysis: Is it an informative and meaningful analysis? Gen Psychiatry. 2019;32(4):3–6.

**The Editor commented:**

In response to my question 2, you stated that: “We selected to test study hypotheses with students because we could access and track this population; however, it is worth noting that this age group – representing emerging adulthood – have been the focus of past research which has found robust links between self-determined motivation and well-being in solitude...”

While I find your rationale sensible, it doesn't account for the fact that students may be a poorly generalizable population in which to address the study questions. I recommend that you acknowledge the limitations of college student sampling in your General Discussion.

**Response:** I have now strengthened this point in the General Discussion on page 28: “A third limitation is that both studies relied on college or university samples. In this research, we recruited participants within the typical age range of those who attend universities in the United States, with a mean age of 20 years, lending caution to any generalizations to the larger population beyond young adults who attend higher education in the US. Whether education levels and different life circumstances might contribute to motivation and preference for solitude is an empirical question that warrants future research.”

Finally, **Reviewer 1 commented:**

“1) It's still not clear in the Procedure section of Study 1 what measures or questions were asked in the daily surveys. The initial survey is clearly explained, but I have to infer what was asked in the daily surveys. The statement on p. 14 under "Diary Survey" is a bit vague ("For each diary surveys [sic], participants reported the events that happened to them that day, including their experiences with social and solitary experiences.") and I had to assume that the measures for dispositional autonomy and introversion measures were included in each daily survey, but this was not explicitly stated. 2) Change AFI to IAF on p. 13.”

**Response:** I have added more details to the Procedure section of Study 1 on page 14: “For each diary survey, participants were asked about a significant event that happened to them that day, their experiences of such event (e.g., autonomy, need satisfaction, positive and negative affect), their self-determined motivation for solitude and levels of preference for solitude. Only self-determined motivation for solitude and preference for solitude were the variables of interest for this present paper, whereas other questions were part of a separate project studying the link between daily positive events and later memories of such events.”

For the measures for dispositional autonomy and introversion measures, I believe the reviewer might have missed what I said on page 13: “All participants were enrolled after submitting an initial survey. Included in the initial survey were several personality measures, including the Introversion subscale from the Big-Five Inventory (40) and the Index of Autonomous Functioning (IAF) (27). Descriptions of personality measures used in this study are provided below.”

I hope those revisions are sufficient in addressing the comments. I am looking forward to any further feedback to help improve this manuscript.

Sincerely,

Thuy-vy Nguyen
